# Age Dependent Hypothalamic and Pituitary Responses to Novel Environment Stress or Lipopolysaccharide in Rats

**DOI:** 10.3389/fnbeh.2018.00055

**Published:** 2018-03-19

**Authors:** Sandy Koenig, Janne Bredehöft, Alexander Perniss, Franziska Fuchs, Joachim Roth, Christoph Rummel

**Affiliations:** ^1^Institute of Veterinary Physiology and Biochemistry, Justus Liebig University Giessen, Giessen, Germany; ^2^Institute of Anatomy and Cell Biology, Justus Liebig University Giessen, Giessen, Germany; ^3^Marburg Center for Mind, Brain and Behavior–MCMBB, Philipps-University Marburg, Marburg, Germany

**Keywords:** novel environment stress, lipopolysaccharide, fever, hyperthermia, cage switch, NF-IL6, immune-to-brain communication, aging

## Abstract

Previously, we have shown that the transcription factor nuclear factor interleukin (NF-IL)6 can be used as an activation marker for inflammatory lipopolysaccharide (LPS)-induced and psychological novel environment stress (NES) in the rat brain. Here, we aimed to investigate age dependent changes of hypothalamic and pituitary responses to NES (cage switch) or LPS (100 μg/kg) in 2 and 24 months old rats. Animals were sacrificed at specific time points, blood and brains withdrawn and analyzed using immunohistochemistry, RT-PCR and bioassays. In the old rats, telemetric recording revealed that NES-induced hyperthermia was enhanced and prolonged compared to the young group. Plasma IL-6 levels remained unchanged and hypothalamic IL-6 mRNA expression was increased in the old rats. Interestingly, this response was accompanied by a significant upregulation of corticotropin-releasing hormone mRNA expression only in young rats after NES and overall higher plasma corticosterone levels in all aged animals. Immunohistochemical analysis revealed a significant upregulation of NF-IL6-positive cells in the pituitary after NES or LPS-injection. In another important brain structure implicated in immune-to-brain communication, namely, in the median eminence (ME), NF-IL6-immunoreactivity was increased in aged animals, while the young group showed just minor activation after LPS-stimulation. Interestingly, we found a higher amount of NF-IL6-CD68-positive cells in the posterior pituitary of old rats compared to the young counterparts. Moreover, aging affected the regulation of cytokine interaction in the anterior pituitary lobe. LPS-treatment significantly enhanced the secretion of the cytokines IL-6 and TNFα into supernatants of primary cell cultures of the anterior pituitary. Furthermore, in the young rats, incubation with IL-6 and IL-10 antibodies before LPS-stimulation led to a robust decrease of IL-6 production and an increase of TNFα production by the pituitary cells. In the old rats, this specific cytokine interaction could not be detected. Overall, the present results revealed strong differences in the activation patterns and pathways between old and young rats after both stressors. The prolonged hyperthermic and inflammatory response seen in aged animals seems to be linked to dysregulated pituitary cytokine interactions and brain cell activation (NF-IL6) in the hypothalamus-pituitary-adrenal axis.

## Introduction

Stress is an omnipresent stimulus in animals and humans i.e., an alarm reaction as first outlined by Hans Selye in 1936 (Rochette and Vergely, 2017). Evolutionary conserved mechanisms are known to orchestrate the stress response via the autonomic nervous system and the hypothalamus pituitary adrenal (HPA) axis leading to adjustments in effector organs and preparing the “fight and flight” reaction. Interestingly, this stress response also includes an increase in body core temperature, which as well seems to be a common beneficial mode of action to fight the stressor. Indeed, humans and a variety of animal species including baboons, pigs, rabbits, squirrels, rats, mice (Bouwknecht et al., 2007) or wild living impala (Meyer et al., 2008) show such an increase in core body temperature, namely stress-induced hyperthermia. Again, a variety of stressors can elicit this hallmark of stress reaching from so-called stage fright or in German literally translated “lamp fever,” when giving a speech in front of a big audience or before examinations in humans (Marazziti et al., 1992), to novel environment, restraint, capture, or social defeat in animals (Oka et al., 2001; Bouwknecht et al., 2007; Meyer et al., 2008; Nakamura, 2015). Interestingly, such an increase in body core temperature can persist for weeks to even years when the psychological stress is exposed repeatedly and/or is of high intensity; a scenario like this is termed “psychogenic fever” (Timmerman et al., 1992; Oka et al., 2001; Nakamura, 2015). In a previous clinical study up to half of cases of high body temperature that were not related to any common abnormalities were diagnosed as psychogenic (Nozu and Uehara, 2005). In rats, for example, social defeat stress induces chronic hyperthermia lasting at least 8 days after cessation of the stressor (Hayashida et al., 2010).

Recently, Nakamura and colleagues have revealed important insights into the neural circuit for psychological stress-induced hyperthermia using experimental animal studies (Nakamura, 2015). For example, the medial prefrontal cortex seems to be one higher brain structure that can activate the dorsal portion of the dorsomedial hypothalamus (DMH), which in turn, directly stimulates sympathetic premotor neurons in the rostral medullary raphe region to increase thermogenesis in brown adipose tissue and to decrease heat dissipation via vasoconstriction (Nakamura, 2015). Thus, this process ultimately leads to an increase in body core temperature i.e., hyperthermia. Moreover, the ventral portion of the DMH drives activation of the paraventricular nucleus (PVN), the first step in HPA axis action. In more detail, corticotropin releasing hormone (CRH) is secreted from parvocellular neurons of the PVN via the median eminence (ME) into the portal circulatory system to activate adrenocorticotropic (ACT) cells in the anterior pituitary lobe. These, in turn release ACT hormone (ACTH) into the circulation, which increases plasma levels of glucocorticoids derived from the adrenal cortex (Papadimitriou and Priftis, 2009).

Previously, we used a model of novel environment stress (NES i.e., cage switch) in rats and have shown that nuclear factor interleukin 6 (NF-IL6), a pivotal transcription factor during inflammation in the central nervous system (Ejarque-Ortiz et al., 2007; Damm et al., 2011; Pulido-Salgado et al., 2015; Schneiders et al., 2015), is also activated in brain structures of the HPA-axis including the anterior lobe of the pituitary (Damm et al., 2011; Fuchs et al., 2013). NF-IL6 not only serves as spatiotemporal activation marker during inflammatory and psychological stress but as well appears to be involved in the modulation of the stress response (Rummel, 2016). Indeed, NF-IL6 was activated in ACT cells and seemed to contribute to tumor necrosis factor (TNF)α expression accompanied by inhibition of ACTH release (Fuchs et al., 2013). However, NF-IL6 deficient mice were capable of mounting a normal HPA-axis response, while the circadian rhythm of circulating glucocorticoid levels was disturbed (Schneiders, 2015).

In the aging population of the western world, changes in the stress and HPA-axes are prone to occur; distinct patterns of diurnal cortisol and a lack of social support and emotional regulation capacities might contribute to a disappearing resilience for coping with psychological stress and infectious diseases in older adults (Gaffey et al., 2016). In a previous study, we revealed that fever, the actively controlled hallmark of systemic lipopolysaccharide (LPS)-induced inflammation, is prolonged in aged rats (Koenig et al., 2014). Moreover, using the cage switch paradigm of NES (unpublished observation), we observed a prolonged hyperthermic response in 24 months aged rats compared to their young 2 months old counterparts, although previous reports revealed no age dependent difference (Foster et al., 1992) or even a smaller response to psychological stress in the aged (Wachulec et al., 1997).

Here, we aimed to further investigate the cage switch-induced hyperthermic response in aged compared to young rats and to reveal more insights into age dependent LPS-induced changes in NF-IL6 activation in components of the HPA-axes, namely, the PVN, the ME and the pituitary. Plasma IL-6 levels were analyzed as potential circulating mediator, which was previously reported to increase with NES (LeMay et al., 1990; Soszynski et al., 1996) and is involved in NF-IL6-activation (Akira et al., 1990; Damm et al., 2011). The mRNA expression of IL-6, NF-IL6 and suppressor of cytokines signaling (SOCS)3 were used as inflammatory markers in the brain. SOCS3 acts as a negative regulator of IL-6-signaling, and with some limitations, can be used as indirect marker of its action on the brain (Lebel et al., 2000; Rummel, 2016). Previous reports suggested that prostaglandin E2 (PGE2), a crucial terminal mediator in the induction of the febrile response, might as well be involved in cage stress-induced increase in body core temperature (Kluger et al., 1987; Morimoto et al., 1991). Therefore, the mRNA expression of the rate-limiting enzymes of the prostaglandin synthesis pathway, namely, cyclooxygenase 2 (COX2) and microsomal prostaglandin E synthase (mPGES) were analyzed. In order to detect changes in the mechanisms of HPA-axis activation, we investigated CRH and proopiomelanocortin (POMC), two important factors implicated in the formation of ACTH and the final release of corticosterone. To further gain more information about age related changes of the cytokine network in the pituitary we applied cytokine specific antisera in LPS-stimulated primary cell cultures of the anterior pituitary lobe of aged compared to young rats.

## Materials and methods

### Animals

The study was performed in young (2 months) and old (23–24 months) male Wistar rats (*rattus norvegicus sp*) with a body weight (BW) of 200–250 g (young male), and 748 ± 27,5 g (old male). For measuring core body temperature and motor activity, biotelemetry transmitters (VM-FR TR-3000; Mini-Mitter, Sunriver, OR) were implanted in the abdominal cavity of the rats at least 1 week prior to the experiment. Before surgery, animals were anesthetized using 60 mg/kg ketamine hydrochloride (Albrecht, Aulendorf, Germany) and medetomidin 0.25 mg/kg (CP Pharma Handelsgesellschaft GmbH, Burgdorf, Germany). Meloxicam [5 mg/kg, subcutaneous (s.c.), Boehringer Ingelheim, Ingelheim, Germany] was administered for analgesic treatment pre- and post-surgery. A data acquisition system (Vital View; Mini Mitter) ensured automatic control of data collection and analysis. Body temperature and motor activity were recorded at 5-min intervals. During the total duration of the experiment (3 days before surgery, during the recovery period and for the experimental procedures) rats were housed individually in a temperature- and humidity-controlled climatic chamber (Weiss Umwelttechnik GmbH, Typ 10′US/+5 to +40 DU, Germany) adjusted to 26°C and 50% humidity, with constant access to water and powdered lab chow. Artificial lights were on from 7:00 to 19:00. Animals were accustomed to the handling procedures at least 3 days prior to the experiment. Animal care, breeding and experimental procedures were conducted in accordance with the German animal protection law and the local Ethics committee “Regional Council Giessen” (ethics approval numbers GI 18/2 Nr. 1/2011, GI 18/2 Nr. 51/2008 and V54-19, c20/15c GI18/2).

### Treatment and experimental protocols

#### Experiment 1: age-dependent differences in response to novel environment stress

Acute psychological stress was induced by use of the NES model. Single housed rats were removed from their home cage and quickly placed into a new experimental Plexiglas cage (different to animal facility housing cages and novel for the animals), whereas control animals stayed in their original surroundings. All cages enabled the rats to smell and see their conspecifics in other cages within the climatic chamber. Additional physiological data were gained by recording body temperature and motor activity of the undisturbed animals on the day preceding the experiment, thereby collecting supplementary control data for analysis without sacrificing additional animals. After 90 min, rats were deeply anesthetized with sodium pentobarbital [160 mg/kg, intraperitoneally (i.p.), Narcoren; Merial, Hallbergmos, Germany], blood samples were collected via cardiac puncture and rats were transcardially perfused with 200–300 ml ice-cold 0.9% saline. The time point was chosen according to a previously demonstrated peak of NF-IL6-activation in PVN and the pituitary in young rats (Fuchs et al., 2013). Brains and pituitaries were quickly removed, frozen in powdered dry ice and stored at −55°C until analysis. All experimental procedures were performed between 08:30 and 12:30.

#### Experiment 2: age-dependent differences in LPS-induced HPA-axis activation

On the day of the experiment, rats were injected i.p. with LPS (100 μg/kg BW; derived from *Escherichia coli*, serotype 0128:B12; Sigma-Aldrich, Deisenhofen, Germany). Control animals were injected with an equivalent volume of sterile pyrogen-free 0.9% PBS (1 ml/kg; Dulbecco's phosphate-buffered saline; PAA, D-Cölbe). All injections were performed between 11:00 and 13:00. After 24 h, rats were perfused and sampling of brains and pituitaries was performed as described above. We previously found that NF-IL6-activation in the pituitary peaked 8 h after LPS-stimulation and returned to baseline at the 24 h time point (Fuchs et al., 2013), while LPS-induced fever was maintained over 24 h in aged but not young rats using the same dose and serotype of LPS (Koenig et al., 2014). Here, we chose this time point to investigate if activation patterns of NF-IL6 in brains structures of the HPA-axis may be prolonged in aged rats compared to the young counterparts.

### Tissue processing for immunohistochemistry (IHC) and real-time polymerase chain reaction (RT-PCR)

For IHC, coronal 20 μm brain and pituitary sections were cut on a cryostat (HM 500, Microm, Walldorf, Germany), thaw-mounted on poly-L-lysine-coated glass slides and stored at −55°C. Sections encompassed hypothalamic brain structures implicated in the HPA-axis including the PVN and the ME, as well as the pituitary anterior lobe (AL), intermediate lobe (IL) and posterior lobe (PL) and were prepared using the stereotaxic rat brain atlas of Paxinos and Watson (1998) as reference (Paxinos and Watson, 1998). For RT-PCR analysis, another 10–15 consecutive frozen 80 μm sections containing the hypothalamus (bregma 0.50 to−3.5 mm) were stapled on glass slides; the hypothalamus was dissected and stored at −55°C for RNA-extraction.

### Real-time PCR

Total RNA from the collected hypothalamic tissue sections was extracted with Trizol (Invitrogen, Carlsbad, CA) according to the manufacturer's protocol. Reverse transcription of 1 μg of total RNA was performed using 50 U murine leukemia virus (MULV) reverse transcriptase, 50 μM random hexamers and 10 mM dNTP mix in a total reaction volume of 20 μl (Applied Biosystems, Foster City, CA). Following reverse transcription, quantitative real-time PCR was carried out in duplicate using a preoptimized primer/probe mixture (TaqMan Gene Expression Assay) and TaqMan universal PCR Master Mix (Applied Biosystems) on a StepOnePlus Real-Time PCR System (Applied Biosystems). For normalization of cDNA quantities between different reactions, house-keeping gene β-actin (catalog No. 4352340E; Applied Biosystems) was measured as a reference, as its stability during aging and inflammation in the brain had been confirmed in previous experiments (Koenig et al., 2014). The relative expression is presented using the 2-(ΔΔCt) method as previously used (Koenig et al., 2014) and described in more detail (Dangarembizi et al., 2018). The sample values represent x-fold differences from a control sample (given as a designated value of 1) within the same experiment. Assay IDs for the analyzed genes are as follows: IL-6 (Rn01410330_m1); COX-2 (Rn00568225_m1); CRH (Rn01462137_m1); POMC (Rn00595020_m1); NF-IL6 (Rn00824635_s1); SOCS3 (Rn00585674_s1).

### Immunohistochemistry

Frozen brain and pituitary sections were briefly air-dried at room temperature (RT) for 7 min and then immersion-fixed in 2% paraformaldehyde (Merck, Darmstadt, Germany) diluted in PBS for 10 min at RT. After three consecutive washes in PBS, the sections were incubated at RT for 1 h with a blocking solution consisting of PBS, containing 10% normal donkey serum (NDS; Biozol, Eching, Germany) and 0.1% triton X-100 (Sigma-Aldrich). Double IHC was performed in order to determine NF-IL6 immunoreactivity in specific cell populations. The primary antibody (AB) rabbit anti NF-IL6 polyclonal IgG (1:5000; cat. Sc-150; Santa Cruz Biotechnology, CA, USA) was applied in conjunction with an additional AB to detect specific cell marker proteins. As such, the rabbit anti NF-IL6 polyclonal IgG was either combined with sheep anti von Willebrand (vW) polyclonal IgG (1:3000; cat. SARTW-IG; Affinity Biologicals, Ancaster, Canada) to stain for endothelial cells or with the mouse anti glial fibrillary acidic protein (GFAP) polyclonal IgG (1:2000; cat. MAB3402; Millipore, Billerica, MA, USA) to detect astrocytes or pituicytes. In additional sets of sections, rabbit anti NF-IL6 polyclonal IgG was used together with mouse anti cluster of differentiation (CD) 68 monoclonal IgG (1:500; cat. MCA341R; AbD Serotec; Oxford, UK) to determine potential colocalization of NF-IL6 signals in brain immune cells. Sections were incubated with a combination of these ABs for 20–22 h at 4°C, followed by three consecutive washes in PBS and visualization with Cy3-conjugated anti-rabbit IgG (1:500; cat. 711-165-152; Jackson Immuno Research, West Grove, PA, USA) for NF-IL6 and Alexa-488-conjugated anti-sheep or anti-mouse IgG for cell type markers (1:500; cat. AZA11015 or AZA21202 respectively, MoBiTec GmbH, Göttingen, Germany) after a 2-h incubation at RT. Sections were counterstained with the nuclear 4.6-diamidino-2-phenylindole (DAPI, 1:1000 dilution in PBS) stain (Mobitec GmbH, Göttingen, Germany) to demonstrate nuclear localization of NF-IL6 immunoreactivity (IR). In order to prevent distracting signals from autofluorescent lysosomal storage bodies that accumulate in many tissues during aging, sections from old animals were additionally treated for 5 min with Autofluorescence Eliminator Reagent® (Merck Millipore, Schwalbach, Germany), followed by two consecutive washes in 70% ETOH (Merck, Darmstadt, Germany) diluted in PBS and two final washes in PBS. Finally, all sections were coated with a glycerol/PBS solution (Citifluor, LTD, London, UK), coverslipped (glass cover slips) and stored at 4°C until microscopic analysis was performed. For control experiments, primary ABs were replaced by species-specific antisera to detect any cross reactivity or unspecific binding. Specificity of the signals of all primary ABs has been confirmed in previous experiments (Damm et al., 2011).

Further information with regard to primary ABs: “NF-IL6: Specificity of the NF-IL6 AB was previously confirmed by preabsorption with the respective blocking peptide (sc-150 P, Santa Cruz) and staining is absent in NF-IL6-deficient animals (Ejarque-Ortiz et al., 2007; Schneiders et al., 2015). Staining patterns confirm previous studies using *in situ* hybridization of NF-IL6 mRNA expression in the mouse brain (Nadeau et al., 2005). The AB was raised against the C-terminus of the protein and recognizes the appropriate bands by western blot (Ejarque-Ortiz et al., 2007; Damm et al., 2011).

Von Willebrand factor: The AB is typically applied to stain endothelial cells with a characteristic morphology and distribution and has been previously used in mice, guinea pigs, and rats (Yamamoto et al., 1998; Konsman et al., 2004; Rummel et al., 2005, 2008). Again, patterns visualized by *in situ* hybridization of vW mRNA expression confirm specificity of labeling (Yamamoto et al., 1998).

Glial fibrillary acidic protein: The cytoplasmatic class III intermediate filament GFAP is broadly used to stain astrocytes in several species including the rat [e.g. Debus et al., 1983; Rummel et al., 2005]. A single band is detected at 51 kDa by Western blot in total brain lysates (manufacturer's data sheet).

Cluster of differentiation 68 (CD68/ED1): This monoclonal mouse AB is raised against rat spleen cells and is commonly applied to detect activated microglia (Bauer et al., 1994; Wuchert et al., 2009), perivascular cells (Graeber et al., 1989) and/or phagocytosing macrophages (Bauer et al., 1994) in rat or mouse brain sections as well as primary glial cultures, which show *de novo* synthesis of the CD68 (Bauer et al., 1994; Damoiseaux et al., 1994).

### Microscopical analysis

A conventional light/fluorescent Olympus BX50 microscope (Olympus Optical, Hamburg, Germany) with a black and white Spot Insight camera (Diagnostic Instruments, Visitron systems, Puchheim, Germany) was used for analyzing the sections and taking images. By means of image editing software (MetaMorph 5.05) the individual images were combined into red/green/blue color figure plates, brightness and contrast were adjusted and the images stored as TIFF files (Adobe Photoshop 5.05). All sections were processed the same way to enable comparison. Semiquantitative or quantitative evaluation of the targeted sections was performed directly for each experiment either by estimates of density or by counting respective signals. For the first method, a five-point scale was used for rating: –(1) no signals, ± (2) single signals in some cases, + (3) low density; ++ (4) moderate density, +++ (5) high density. 2–3 sections per animal and brain or pituitary structure were evaluated and averaged for each animal and subsequently for each group (means of the means). The second method consisted in counting all nuclear NF-IL6 signals and the total number of DAPI-positive cells of the analyzed brain or pituitary structure (3 – 16 sections per animal). After averaging these data for each animal, the percentage of NF-IL6 positive cells out of DAPI-positive cells was calculated for each group.

### Primary cell culture of the anterior lobe of the pituitary

As previously described (Fuchs et al., 2013), primary cell cultures of the anterior lobe of the rat pituitary were established from young and old male rats. In more detail, previous publications dealing with primary cell cultures of the anterior pituitary lobe (Crack et al., 1997; Carretero et al., 2003; Lee et al., 2008) were used to establish the present protocol. Moreover, cell culture conditions were adjusted to primary neuro-glial cell cultures of the circumventricular organs [e.g., (Ott et al., 2010)]. The present culture contains 5% ACTH immunoreactive cells and S100 immunoreactive folliculostellate cells, as to be expected for a cell culture of the anterior pituitary lobe (Fuchs et al., 2013). For each experiment, the relative cell density was checked to confirm that even when plating the same amount of cells conditions were stable in between sets of experiments. Although these cultures have been extensively used in the past and are well established, a full characterization of all present cell phenotypes is difficult and has not been performed representing also a limitation of the present study. Two to three animals were sacrificed, quickly decapitated with a guillotine for each preparation, and the heads were immersed in ice-cold 0.1 M phosphate-buffered saline, pH 7.4 (PBS; PAA Laboratories GmbH, Coelbe, Germany). The pituitaries were quickly removed under aseptic conditions and transferred in a Petri dish containing ice-cold oxygenated Earle's Balanced Salt Solution (EBSS; Invitrogen, Darmstadt, Germany). Thereafter, the anterior lobe of the pituitary was dissected and placed in a Petri dish with ice-cold, oxygenated Hanks Balanced Salt Solution (HBSS) devoid of Ca^2+^ and Mg^2+^ (Biochrom, Berlin, Germany) and supplemented with 20 mM HEPES (Sigma-Aldrich), pH 7.4. The tissue was treated for 90 min at 37°C in a solution of 2 mg/ml dispase-1 (Roche Diagnostics, Mannheim, Germany) in oxygenated HBSS with 20 mM HEPES, pH 7.4. After enzymatic treatment, the pituitary fragments were washed once with HBSS containing 1.0 mM EDTA (Sigma-Aldrich) to inactivate the enzyme, followed by three washes with complete medium, composed of Dulbecco's Modified Eagle Medium (DMEM; Invitrogen, Darmstadt, Germany) supplemented with 10% Fetal calf serum (FCS, PAA Laboratories GmbH, Coelbe, Germany), penicillin (100 U/ml) streptomycin (0.1 mg/ml) and 4mM L-glutamine (Biochrom AG, Berlin, Germany). By repeated trituration with a 1 ml Eppendorf pipette tip, the tissue was mechanically dissociated in 2 ml complete medium. The cell number was determined using an improved Neubauer C-Chip (NonoEnTek, Seoul, Korea) and, after dilution to ~250,000 cells per ml, the cells were plated onto prewarmed glass coverslips (MAGV GmbH, Rabenau, Germany) coated with poly-L-lysine (0.1 mg/ml; Biochrom AG, Berlin, Germany), which formed the bottom of a reusable Flexiperm-micro-12 well (6 mm diameter, Greiner Bio-One GmbH, Solingen, Germany) in order to ensure sufficient cell density despite limited absolute cell number. Cells were cultured in a humidified atmosphere of 5% CO_2_ and 95% room air at 37°C. The next day, the medium was exchanged to remove cellular debris. In order to prevent potential stimulatory effects of the FCS, the medium was exchanged with serum-free culture medium after 2 days and experimental treatment procedures were carried out the next day. Cells were incubated with LPS (100 μg/ml) or PBS in serum-free culture medium for 6 h. In addition, cells were pretreated with ABs against the cytokines IL-6 or IL-10 (28.5 μl/ml) (NIBSC, Potters Bar, UK) or solvent (serum-free culture medium) for 30 min before LPS or control stimulation. Bulk ion exchange was used to purify sheep IgG from the crude sheep IL-6 and IL-10 antisera and were a gift by GN Luheshi (Douglas Mental Health University Institute, McGill University, Canada). Application as coating ABs for ELISA enabled to check cross reactivity and specificity for these ABs (Rees et al., 1999; Rummel et al., 2006; Pohl et al., 2009; Harden et al., 2014; Koenig et al., 2014). The supernatants were collected and stored at −55°C for cytokine analysis. Cell culture conditions and treatment protocols (6 h time interval and LPS-dosage) were chosen according to the results of prior experiments performed in-house, which have previously shown to induce a robust increase in IL-6 and TNFα in the supernatant (Wuchert et al., 2008; Fuchs et al., 2013).

### Measurement of cytokines and corticosterone

Cytokine analysis was performed on blood plasma samples and supernatants from pituitary anterior lobe cell cultures. IL-6 and TNFα levels were determined by means of bioassays based on a dose-dependent growth stimulation of IL-6 on the B9 hybridoma cell line and on a cytotoxic effect of TNFα on the mouse fibrosarcoma cell line WEHI 164 subclone 13 as previously reported (Damm et al., 2012). The bioassays showed detection limits of 3 IU IL-6/ml and 6 pg TNFα/ml. Corticosterone levels were analyzed in blood plasma samples using a commercial ELISA kit (ELISA; DRG Instruments GmbH, Marburg, Germany; EIA-4164) according to the manufacturer's instructions. The detection range was 1.13 – 415.75 ng corticosterone/ml.

### Data analysis

For analysis of abdominal temperature, delta T (ΔT) was calculated as temperature of each time point subtracted by the mean temperature from the time interval −120 to 0 min before onset of the novel environment experiment. Abdominal temperatures were analyzed using a three-way repeated measures ANOVA with the between subjects factor age and treatment and the within subjects factor time. Data were divided into 30 min-intervals for analysis and Bonferroni-correction for multiple comparisons was performed, followed—in case of a significant interaction—by a Tukey *post hoc* test (Statistica 12, StatSoft Europe, Hamburg, Germany). Cumulative data on motor activity, relative expression of IL-6, COX2, POMC, CRH, NF-IL6, and SOCS3 in the hypothalamus, blood plasma concentration of cytokines and corticosterone, were analyzed by two-way ANOVA with age (young vs. old) and treatment (stress vs. control) as between-subject factors (Prism 5 software; GraphPad, San Diego, CA). Given a significant interaction, Bonferroni *post hoc* tests were conducted. The cytokine measurements of cell culture supernatants were analyzed separately for each age group by ANOVA followed by Newman-Keuls multiple comparison *post hoc* tests. NF-IL6 positive cells counts were compared separately for each group by parametric *T*-test. *P* values < 0.05 were deemed statistically significant. Data within the small but highly controlled sample size showed moderate homogenous distribution as expected for a biological system of this matter. D'Agostino & Pearson omnibus normality test (omnibus K2, Prism) was applied and revealed that temperature data for each time point were overall normally distributed as well as cumulative motor activity. Homogeneity of variance in primary cell cultures of the anterior pituitary lobe was lower. Due to the rather low n-number D'Agostino & Pearson omnibus normality test was not applicable in other cases. In our hands, the aim of the study was to detect rather large effect sizes. Overall, we believe even with this limitation, our clear and strong effects that became significant are biologically meaningful and supported by the literature. Outlier testing was applied to exclude any data that was outside of an acceptable variance (graphical). All data are presented as mean ± SEM.

## Results

### Influence of aging on stress-induced hyperthermia and on the expression of inflammatory and stress mediators

**Figure 1:**
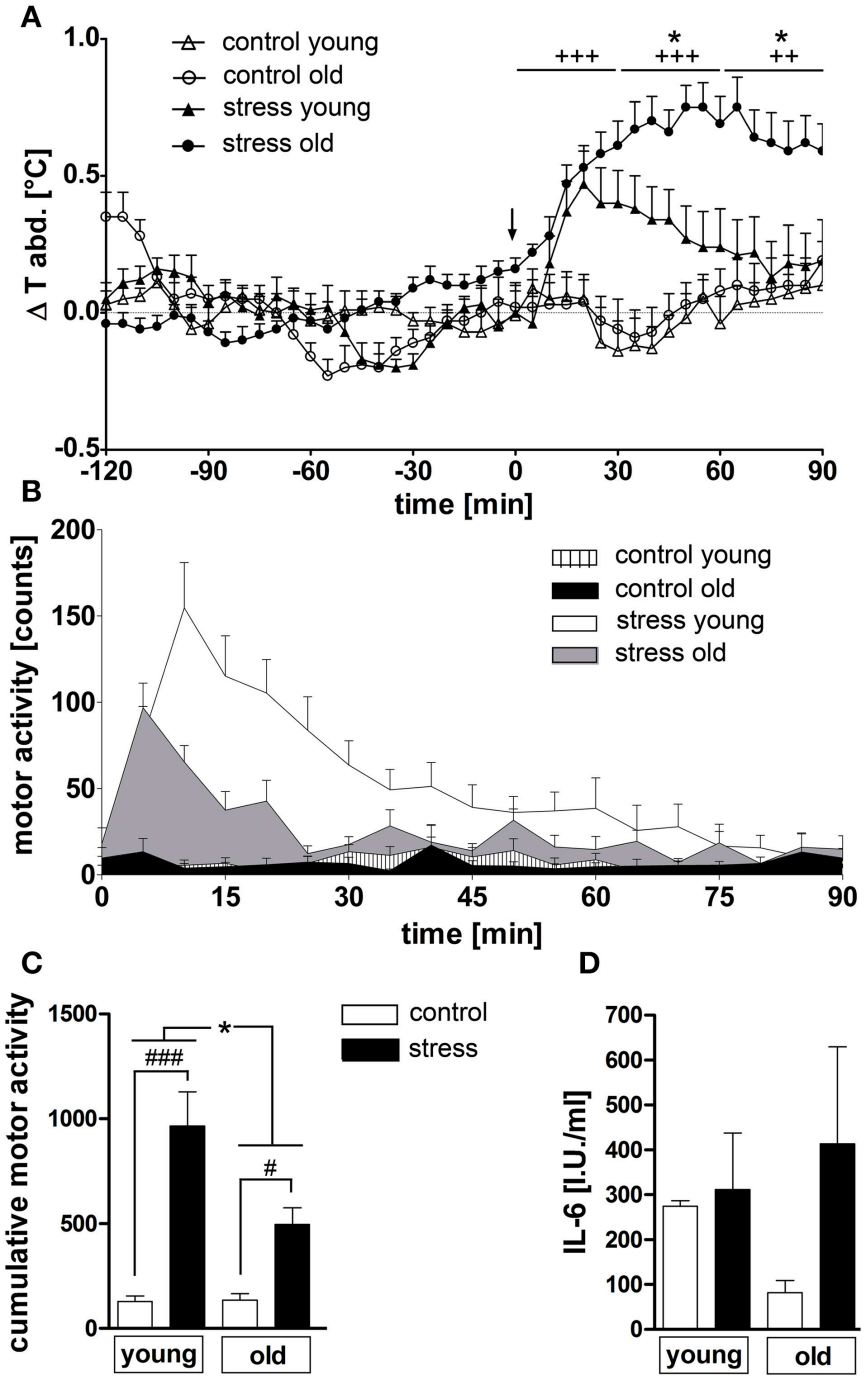
**(A)** Novel environment stress (beginning of stress indicated by a black arrow) over 90 min induced a rise in abdominal temperature in both age groups, which was strongly enhanced in old male rats compared to young male counterparts. **(B,C)** On the contrary, the stress-induced rise in motor activity, present in both age groups, was significantly higher in the young rats. **(D)** Plasma interleukin 6 (IL-6) levels were not affected by treatment or age. + main effect of treatment (not indicated for **(C)**; ^*^ main effect of age; ^#^*post hoc* stress different from control; #, ^*^P < 0.05, ++ *P* < 0.01, +++, ^*###*^*P* < 0.001. Control young *n* = 12 (6 in D), stress young *n* = 12 (4 in **D**), control old *n* = 13 (12 in **B,C**, 5 in **D**), stress old *n* = 14 (13 in **B,C**, 5 in **D**).

NES induced a rise in abdominal temperature in both age groups (Figure 1A). While young rats showed a maximal peak in temperature of 0.47 ± 0.12°C at 20 min, old rats reached a considerably higher peak of 0.75 ± 0.08°C at a later time point of 50 min. Overall, hyperthermia was clearly enhanced in the old rats from 35 to 90 min (*P* < 0.05) compared to the young group and was still present at the end of the experiment in the old animals, while the temperature in the young rats had returned to baseline levels at this time point. Furthermore, NES induced an increase in motor activity in both age groups [main effect of treatment *F*_(1, 45)_ = 42.54, *P* < 0.0001], but this effect was significantly more pronounced in the young rats (*post-hoc P* < 0.001) compared to the old group (*post-hoc P* < 0.05) [main effect of age *F*_(1, 45)_ = 6.372, *P* < 0.05; age × treatment interaction *F*_(1, 45)_ = 6,725, *P* < 0.05] (Figures 1B,C). Plasma IL-6 levels showed no stress-dependent regulation at this time point, independent of age (Figure 1D).

**Figure 2:**
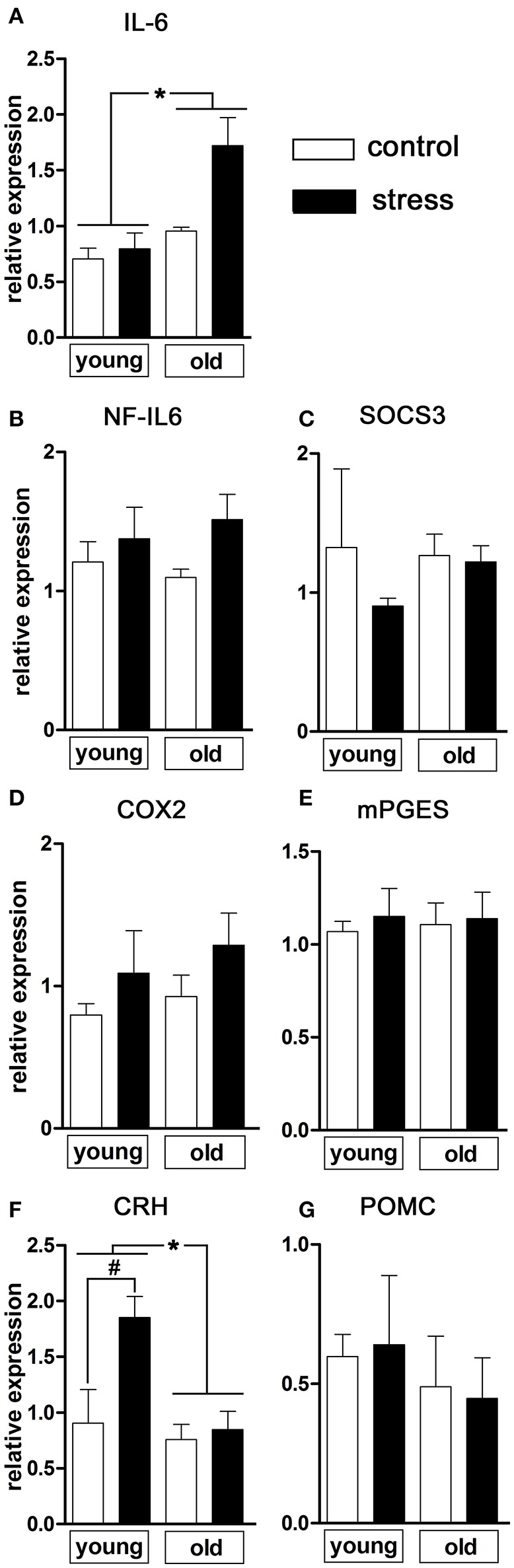
mRNA expression in the hypothalamus of pro-inflammatory mediators and markers of HPA axis activation 90 min after induction of novel environment stress or in unstressed controls in young and old male rats. **(A)** Stress induced an enhanced expression of interleukin (IL)-6 only in the aged rats. **(F)** The expression of CRH (corticotropin releasing hormone), on the contrary, was significantly increased after psychological stress only in the young rats while remaining unchanged in the aged group. (**B–E,G**) The other investigated parameters NF-IL6 (nuclear factor interleukin 6), SOCS3 (suppressor of cytokine signaling 3), COX2 (cyclooxygenase-2), mPGES (microsomal prostaglandin E synthase) and POMC (proopiomelanocortin) were not affected by treatment or age. Main effect of treatment not indicated; ^*^main effect of age; ^#^*post-hoc* stress different from control; ^*^, ^#^*P* < 0.05. Control young *n* = 4, stress young *n* = 4, control old *n* = 5, stress old *n* = 5.

Next, we analyzed a range of relevant inflammatory mediators and markers of HPA-axis activation in order to further investigate the influence of psychological stress on central signaling pathways involved in the manifestation of the febrile response as well as potential age-related differences (Figure 2). Analysis of hypothalamic tissue via RT-PCR showed an age-dependent difference in the expression of the inflammatory cytokine IL-6 [main effect of age *F*_(1, 14)_ = 13.02, *P* < 0.01; main effect of treatment *F*_(1, 14)_ = 6.833, *P* < 0.05], revealing that NES led to an upregulated IL-6 expression only in the old rats (Figure 2A).

With regard to inflammatory transcription factors, we analyzed the expression of NF-IL6 and of SOCS3 as negative regulator and activation marker for the transcription factor STAT3 (Lebel et al., 2000) (Figures 2B,C). In the present experimental paradigm, neither treatment nor aging affected the expression of these factors.

The rate-limiting enzymes of the prostaglandin synthesis pathway COX2 and mPGES remained unaffected by the experimental procedures (Figures 2D,E) suggesting that brain derived PGE2 was not involved in stress-induced hyperthermia in our study.

Regulation of hypothalamic CRH expression by psychological stress showed age-dependent differences [main effect of age *F*_(1, 14)_ = 8.469, *P* < 0.05; main effect of treatment *F*_(1, 14)_ = 6.905, *P* < 0.05; age x treatment interaction F_(1, 14)_ = 4.683, *P* < 0.05] (Figure 2F). While the expression of CRH remained unchanged in old rats (*post-hoc P* > 0.05), young rats showed a significant upregulation of CRH expression after NES (*post-hoc P* < 0.05). No significant changes were detected in POMC expression (Figure 2G).

**Figure 3:**
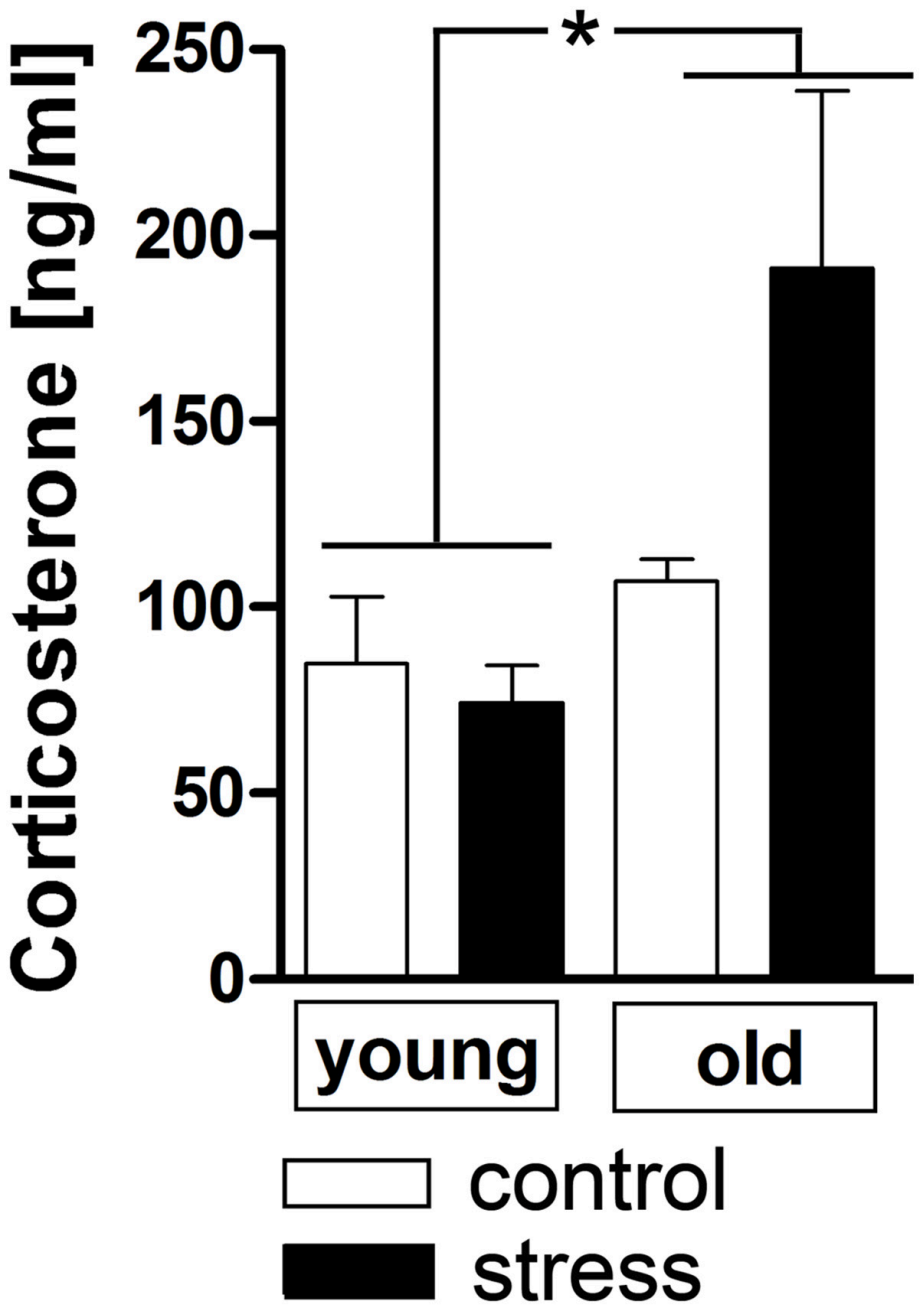
Plasma corticosterone levels after 90 min in stressed and unstressed young and old male rats. Aged rats displayed increased corticosterone levels after psychological stress, while the plasma levels of young rats remained unchanged. ^*^main effect of age; ^*^P < 0.05. Control young *n* = 7, stress young *n* = 4, control old *n* = 5, stress old *n* = 5.

Additionally to the hypothalamic expression of these two factors, corticosterone plasma levels were analyzed as a complementary terminal readout of HPA-axis activity. Contrary to the previous results for hypothalamic CRH expression, aging upregulated the stress-induced secretion of plasma corticosterone [main effect of age *F*_(1, 17)_ = 6.819, *P* < 0.05] (Figure 3).

### NF-IL6 IR is upregulated in the pituitary after psychological stress in old rats

**Figure 4:**
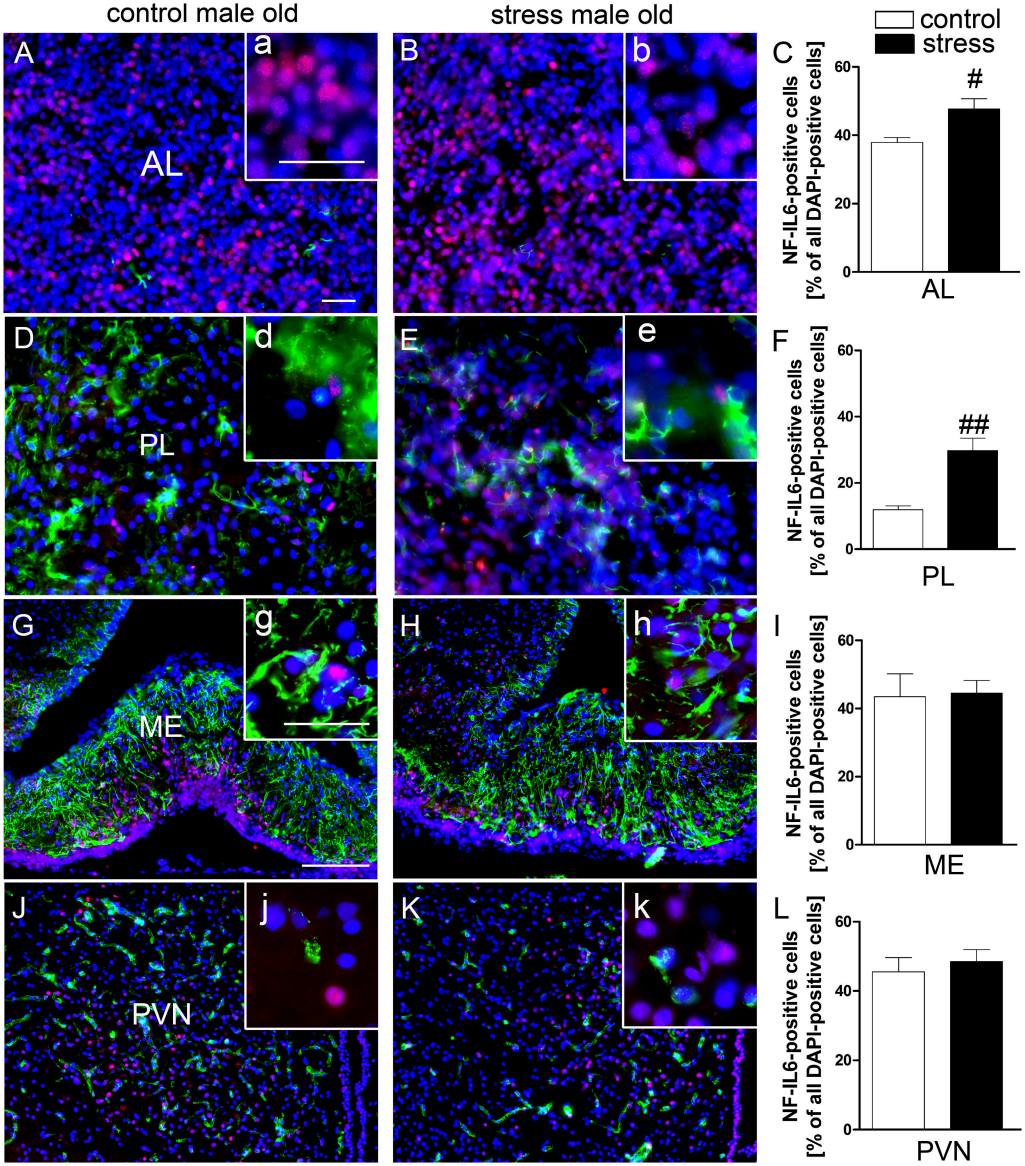
Novel environment stress induced enhanced NF-IL6 (nuclear factor interleukin 6) immunoreactivity (IR, red) in the anterior (AL) and posterior (PL) pituitary lobe **(B,E)** in aged male rats, which was confirmed by further quantitative evaluation (counting of NF-IL6 positive cells) of the immunohistochemistry **(C,F)**. In the median eminence (ME) **(G)** and paraventricular nucleus (PVN) (J) unstimulated controls showed substantial basal NF-IL6 activation. No significant further enhancement of NF-IL6 IR could be observed after novel environment stress **(H,K)** by immunohistochemistry and counting of NF-IL6 positive cells **(I,L)** compared to control animals in these regions. **(A,B,D,E)** GFAP (glial fibrillary acidic protein, green) or **(G,H,J,K)** vW (von Willebrand factor, green) was used to better visualize the pituitary or hypothalamic structures. Cell nuclei were labeled with DAPI (blue). The scale bar in **(A)** represents 25 μm and applies to **(A,B,D,E)**, the scale bar in G represents 100 μm and applies to **(G,H,J,K)**. For all insets (a–k) the scale bar is 25 μm. # stress vs. control; ^#^*P* < 0.05; ^*##*^*P* < 0.01. Control old *n* = 5, stress old *n* = 5 (*n* = 4 for AL and PL); the mean of 2–3 sections of each animal of the mean for each group was used for quantification.

Novel environment stress upregulated NF-IL6 IR in both the anterior and posterior pituitary lobe in old male rats (Figures 4B,E). A low basal NF-IL6 activation was already present in unstressed controls (Figures 4A,D). These qualitative observations were verified by NF-IL6 cell count (represented as percentage of NF-IL6 positive cells out of all DAPI-positive cells), which clearly confirmed that stressed rats showed significantly increased numbers of NF-IL6 positive cells in the anterior (*P* < 0.05) and posterior (*P* < 0.01) lobes (Figures 4C,F). In the ME and the PVN, unstressed control rats showed a high basal activation of NF-IL6 IR (Figures 4G,J). Novel environment stress did not lead to any further significant upregulation of NF-IL6 positive cells in the old rats (Figures 4H,K), which was also affirmed by cell counts (*P* > 0.05) (Figures 4I,L).

### Age-dependent differences in LPS-induced NF-IL6 IR and CD68 IR in the hypothalamus and pituitary

**Figure 5:**
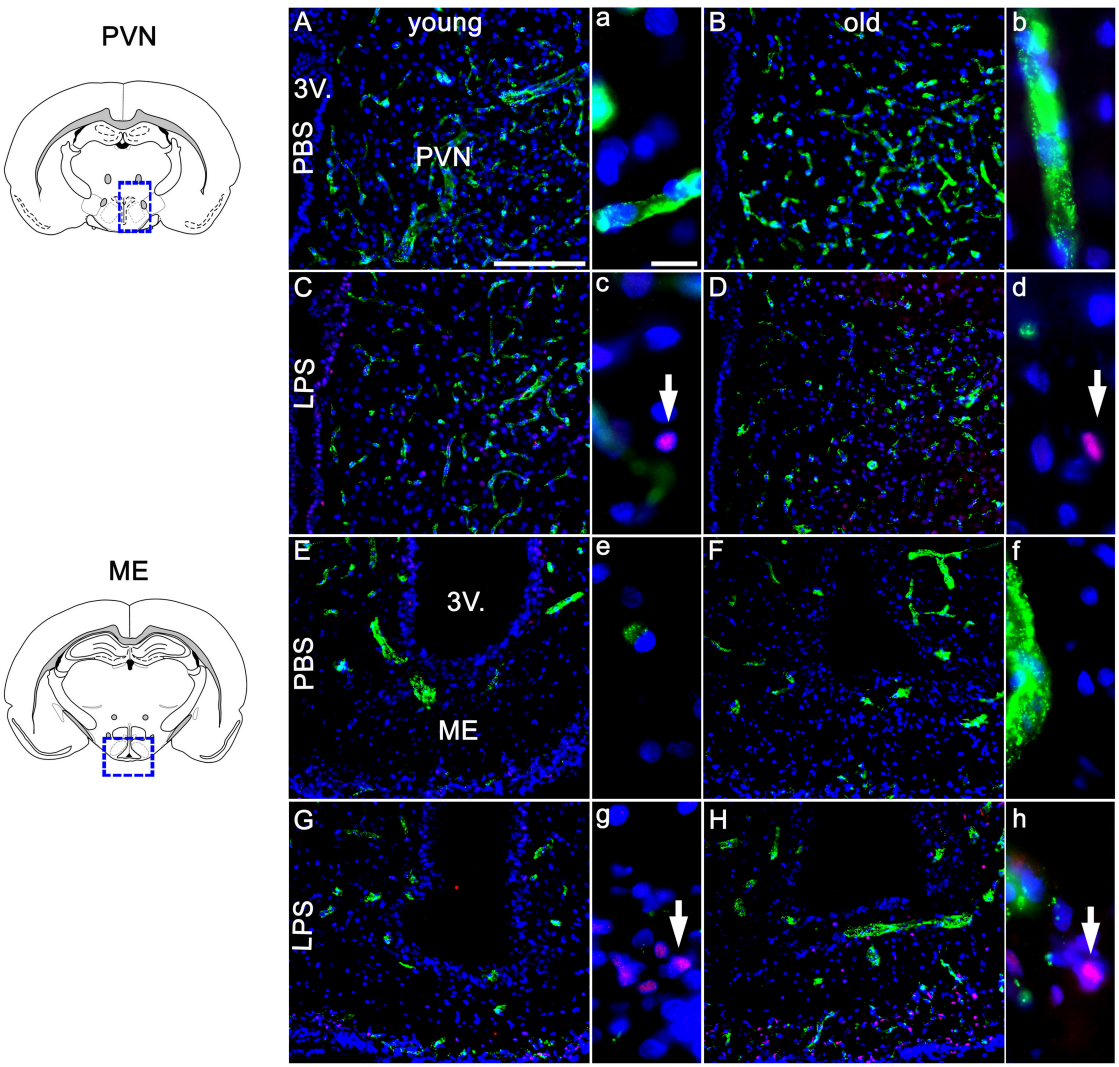
Twenty four hours after LPS-treatment (100 μg/kg), the paraventricular nucleus (PVN, **A–D**) showed scarce activation of NF-IL6 (nuclear factor interleukin 6) signals in isolated cells in young and old rats **(C,D**), without a clear age-dependent distinction. In the median eminence (ME, **E–H**) however, LPS injection led to a considerably higher expression of NF-IL6 immunoreactivity in the aged group **(H)**, as compared to the young counterpart **(G)**. PBS-injected controls showed no NF-IL6 activation in any group or hypothalamic region. vW (green) was used to better visualize the hypothalamic structures. Cell nuclei were labeled with DAPI (blue). White arrows show nuclear NF-IL6 signals. The scale bar in A represents 100 μm and applies to all overview pictures, the scale bar in a equals 10 μm and applies to all insets. Representative pictures from 3–4 sections per animal; PBS old *n* = 5 for ME, 6 for PVN; LPS old *n* = 5 for ME and PVN; PBS young *n* = 5 for ME, 4 for PVN; LPS young *n* = 5 for ME and PVN.

Based on previous research findings (Fuchs et al., 2013), we aimed to perform a closer investigation of potential age-related differences in LPS-induced activation of NF-IL6 in the brain and HPA-axis. Immunohistochemical analysis of NF-IL6 was performed in the PVN and the ME, because they represent important brain structures implicated in the signal transfer within the HPA-axis. In the PVN, LPS-injection induced only a slight increase in NF-IL6 IR irrespective of age (Figures 5A–D). In the ME, a high upregulation of NF-IL6 IR was detected in the old LPS-injected rats (Figures 5F,H), while the young group showed only a minor activation (Figures 5E,G). An additional semi-quantitative five-point scale evaluation was performed for each group and brain structure and confirmed these qualitative observations (Table 1).

**Table 1:** Semi-quantitative analysis of the amount of nuclear nuclear factor interleukin 6 (NF-IL6) signals in the paraventricular nucleus (PVN) and the median eminence (ME) of young and old male rats 24 h after injection of LPS (100 μg/kg) or PBS.

**Brain structure**	**Nuclear NF-IL6 immunoreactivity**			
	**PBS young**	**LPS young**	**PBS old**	**LPS old**
PVN	− (1,25)	± (2,4)	− (1,17)	± (2,2)
ME	− (1,4)	+ (2,8)	− (1,4)	++ **(4)**

*A five-point scale was used to rate the data: − (1) no signals, ± (2) single signals in some cases, + (3) low density; ++ (4) moderate density, +++ (5) high density; n = 3-6 animals per group, 2 sections per animal; the robust difference between young and old is highlighted in bold*.

**Figure 6:**
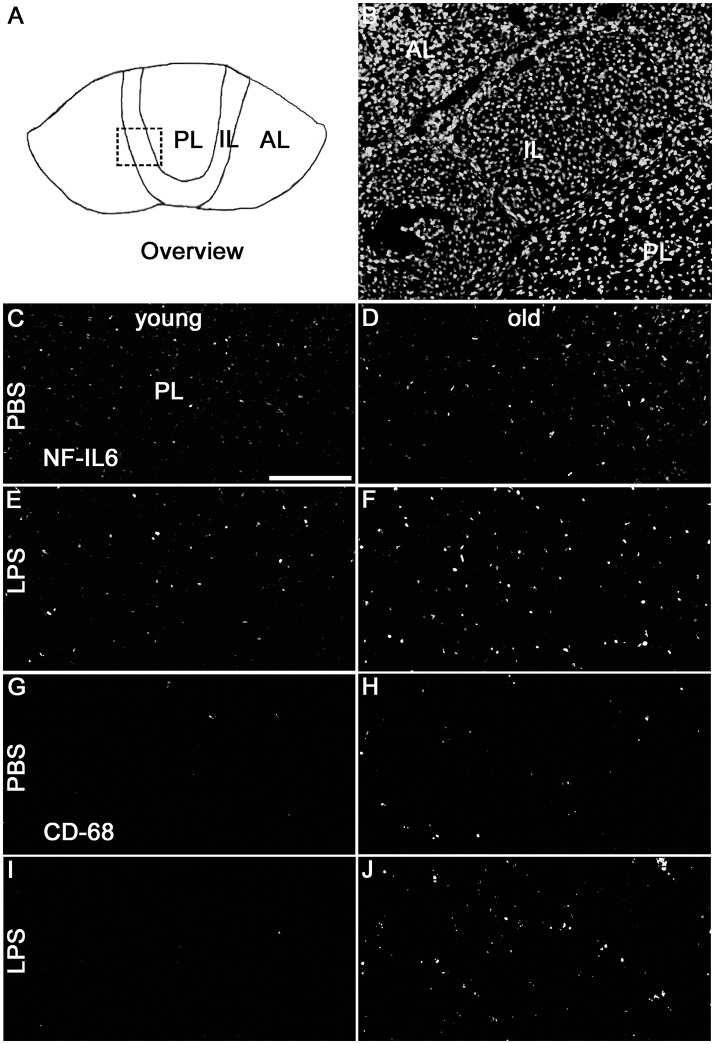
**(A)** The schematic overview illustrates the structure of the pituitary; the dashed square indicates the substructure represented by DAPI-staining in **(B)**, depicting all pituitary lobes [anterior lobe (AL), intermediate lobe (IL), posterior lobe (PL)]. Old control rats **(D)** displayed a higher basal NF-IL6 (nuclear factor interleukin 6) immunoreactivity (white) compared to young controls **(C)**. After LPS-treatment (100 μg/kg), old rats **(F)** showed highly increased NF-IL6 signals, while young rats **(E)** remained unaffected. CD (cluster of differentiation) 68 immunohistochemistry presented a similar result, with increased numbers in CD68 positive cells (white) in old PBS-injected controls **(H)** and strongly enhanced signals in old rats after LPS-injection **(J)**, while young rats showed only isolated positive cells in both cases **(G,I)**. The scale bar in C represents 100 μm and applies to all overview pictures. Representative pictures from 4-10 sections for NF-IL6 and 2 sections for CD68 per animal; PBS and LPS old *n* = 3, PBS and LPS young *n* = 2.

Analysis of the complete pituitary structure (anterior, intermediate and posterior lobe; Figures 6A,B) revealed that NF-IL6 IR was increased in both age groups after LPS treatment in the posterior lobe and remained unchanged in the intermediate lobe (Supplementary Figure 1). In contrast, inspection of the posterior lobe evidenced an increased NF-IL6 upregulation in the aged group (Supplementary Figure 1B). Closer examination of the posterior lobe revealed that aging did not only increase LPS-induced NF-IL6 IR, but even in the PBS-treated aged control group a higher basal NF-IL6 profile compared to the young counterpart was detected (Figures 6C–F). These results were supported by further semi-quantitative evaluation (Table 2). Based on these findings, co-localization studies with GFAP and CD68 were performed in order to investigate if increased NF-IL6 activation is connected to a specific cell population, namely pituicytes or CD68 positive immune cells. While no NF-IL-6 activated pituicytes could be detected (Supplementary Figures 2A–D), NF-IL6 signals were seen in CD68 positive cells in all groups (Supplementary Figures 2E-H). Closer analysis of CD68 cells revealed that aged rats showed an increased number of CD68 positive immune cells compared to young rats under basal conditions (Figures 6G,H), which was significantly upregulated after LPS-treatment in the aged group (Figures 6I,J; see Table 2 for semi-quantitative analysis).

**Table 2:** Semi-quantitative analysis of the number of nuclear nuclear factor interleukin 6 (NF-IL6) signals and CD (cluster of differentiation) 68 positive cells in the anterior (AL), intermediate (IL) and posterior (PL) lobe of the pituitary in young and old male rats 24 h after injection of LPS (100 μg/kg) or PBS.

**Pituitary lobe**	**PBS young**	**LPS young**	**PBS old**	**LPS old**
**Nuclear NF-IL6 immunoreactivity**				
Anterior lobe	+ (2,7)	++ (3,9)	+ (2,73)	++ (3,87)
Intermediate lobe	± (1,5)	± (1,65)	± (1,67)	± (2,2)
Posterior lobe	± (2,3)	++ (3,73)	++ **(3,5)**	+++ **(4,87)**
**CD68 immunoreactivity**				
Anterior lobe	++ (3,5)	++ (4)	++ (3,83)	++ (4,3)
Intermediate lobe	− (1)	− (1)	± (1,83)	± (1,67)
Posterior lobe	± (2,25)	+ (3)	++ **(4)**	+++ **(4,83)**

*A five-point scale was used to rate the data: − (1) no signals, ± (2) single signals in some cases, + (3) low density; ++ (4) moderate density, +++ (5) high density. NF-IL6 immunoreactivity (IR): 2-3 animals per group, 4–10 sections per animal. CD68 IR: 2-3 animals per group, 2 sections per animals; robust differences between young and old are highlighted in bold*.

### Aging affects the regulation of cytokine interaction in the anterior pituitary lobe

**Figure 7:**
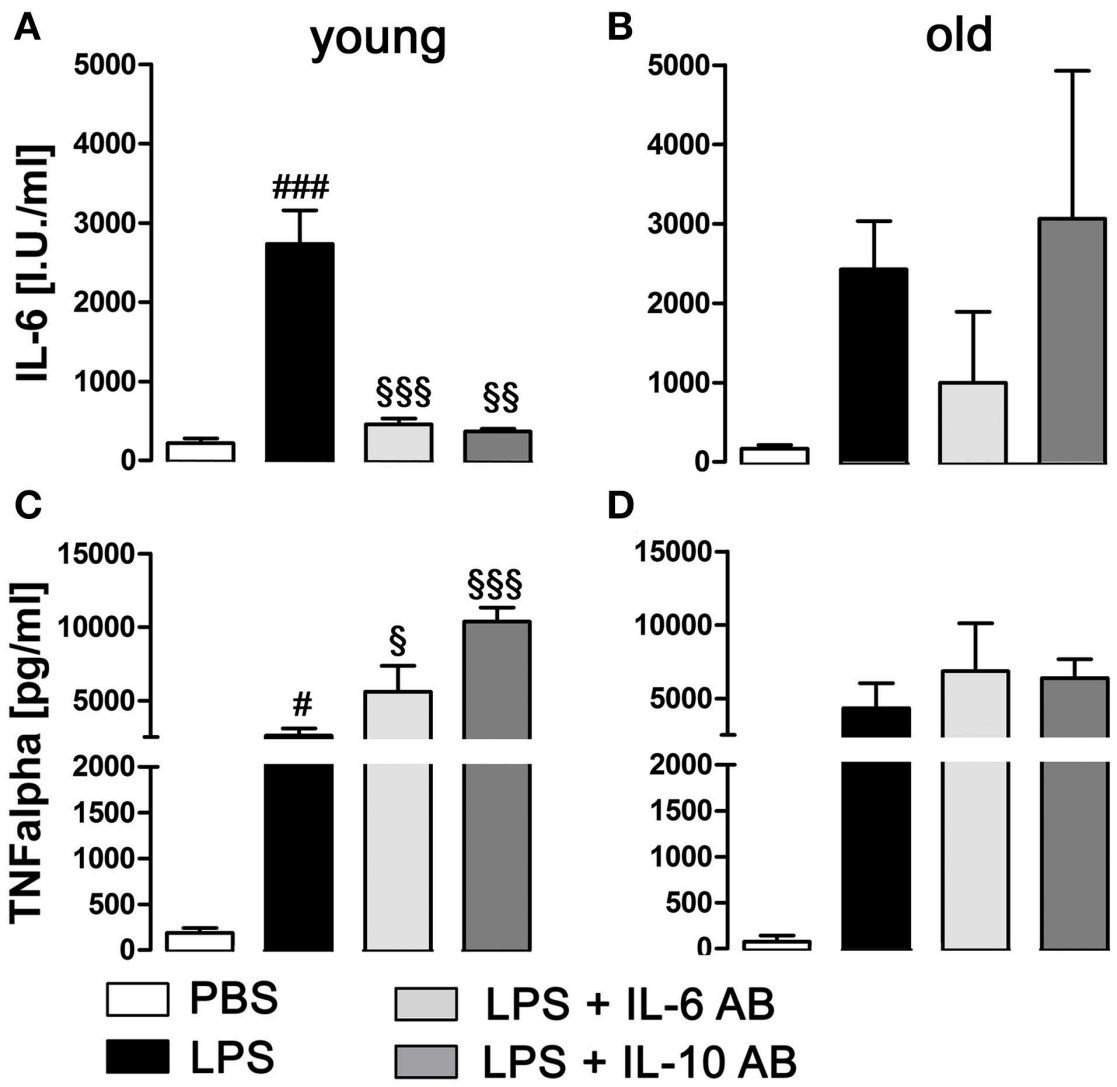
Concentration of tumor necrosis factor alpha (TNFα) and interleukin (IL)-6 in supernatants of primary cell cultures of the anterior pituitary lobe after preincubation with IL-6 or IL-10 antibodies (AB) and/or stimulation with LPS or PBS in young and old male rats. **(A,C)** In young rats, LPS treatment induced a robust increase of IL-6 and TNFα secretion. Preincubation with cytokine ABs decreased the LPS-induced secretion of IL-6 to basal levels, while at the same time increasing TNFα release. **(B,D)** In old rats, there were no significant changes in cytokine secretion, independent of treatment. # LPS vs. PBS; § IL-6 AB/IL10 AB + LPS vs LPS; ^#, §^*P* < 0.05, ^§§^*P* < 0.01; ^*###*, §§§^*P* < 0.001. Data is derived from at least three independent experiments for each treatment.

LPS treatment significantly enhanced the secretion of the cytokines IL-6 (*P* < 0.001) and TNFα (*P* < 0.05) into supernatants by pituitary cells in the young rats (Figures 7A,C), while in the old rats, the increased secretion, although present, was no longer significant for these cytokines (Figures 7B,D). Furthermore, in the young rats, the incubation with IL-6 and IL-10 ABs before LPS stimulation led to a robust decrease of IL-6 production (*P* < 0.001 for IL-6 AB and *P* < 0.01 for IL-10 AB) and an increase of TNFα production (*P* < 0.05 for IL-6 AB and *P* < 0.001 for IL-10 AB) by the pituitary cells. In the old rats, this specific cytokine interaction could not be detected.

## Discussion

In the present study, we are the first to show that NES-induced hyperthermia is prolonged in aged rats when kept at thermoneutral ambient temperature. This response was accompanied by higher IL-6-expression in the hypothalamus as well as circulating corticosterone in aged rats while plasma IL-6 levels did not show any age dependent changes. CRH mRNA expression was increased by NES only in young but not old rats. Similar to our previous study in young rats (Fuchs et al., 2013), cage switch also enhanced NF-IL6-activation in the pituitary of aged rats. Moreover, nuclear NF-IL6 immunoreactivity slightly or robustly increased in both age groups 24 h after LPS-injection in the PVN and the ME, respectively. Interestingly, ME NF-IL6-activation was significantly higher in the old LPS-stimulated rats than in the young counterparts. In addition, pituitary NF-IL6-activation was higher in LPS-stimulated young and old rats; however, a significant basal activity was only detected in the posterior pituitary lobe of old animals. The higher NF-IL6-activity in both the control and LPS-injected aged groups in the posterior pituitary lobe was partly occurring in and was associated with increased numbers of CD68-positive immune cells.

Earlier studies revealed reduced (Wachulec et al., 1997) or unchanged (Foster et al., 1992) NES-induced hyperthermia in aged compared to young rats. Here, we were able to show prolonged NES induced hyperthermia by cage switch in the aged most likely related to the thermoneutral ambient temperature used in the present study. Indeed, aged animals only show a normal (Florez-Duquet et al., 2001; Buchanan et al., 2003; Peloso et al., 2003) or even prolonged febrile response to LPS (Koenig et al., 2014) when kept significantly above (e.g., 26 or 31°C) the subthermoneutral ambient temperature seen and usually recommended for rodent breeding and housing facilities i.e., around 22°C (Gordon, 2012). In young rats, stress-induced increase in body core temperature does not depend on ambient temperature as already reported by Long et al. (1990a) for 24.7°C and 11.1°C, respectively.

Interestingly, NES-induced increase in body core temperature is not a function of increased locomotor activity. Long et al. (1990b) already found that locomotor activity did not correlate with body core temperature. While some contribution of early and quick increase in body core temperature might be related to increased locomotor activity (Fuchs et al., 2013), clearly, the extent of hyperthermia in particular the prolonged response in the aged rats is not linked to it. Old rats actually display significantly lower locomotor activity than young counterparts. In a previous study, we found altered HPA-axis activity in NF-IL6 deficient animals, which was accompanied by prolonged locomotor activity after NES but no concomitant increase in body core temperature (Schneiders et al., 2015). We hypothesized that CRH might be related to these changes (Schneiders et al., 2015) as intracerebroventricular (i.c.v.) injection of CRH has been shown to increase and its inhibition was demonstrated to reduce locomotor activity (Ohata et al., 2002) and body core temperature in response to cage switch (Morimoto et al., 1993). Indeed, NF-IL6 has been previously shown to activate the CRH promotor (Stephanou et al., 1997). In the present study, we found hypothalamic CRH-mRNA expression to be only significantly increased by NES in the young animals while it remained unchanged at basal levels in the aged rats, potentially explaining lower locomotor activity in the aged compared to young animals but not sustained hyperthermia in the old rats exposed to NES.

Overall, these data indicate age-dependent differences between the responsiveness to acute stressors like NES between young and old rats. Possibly, defective stop signals could explain the exaggerated/prolonged hyperthermic response to this acute stressor in the old rats. We found significantly higher corticosterone plasma levels in the aged rats suggesting some resistance related effects for the negative feedback loop of circulating corticosterone on hypothalamic glucocorticoid receptors (Gaffey et al., 2016). It might appear somehow surprising that there was a significant corticosterone increase in plasma of old but not young rats 90 min after NES in the presents study. However, the corticosterone response is rather quick. Previous reports have shown an increase of corticosterone plasma levels in rats already after 10 min open field stress (Morrow et al., 1993) or 60 min after acute pain stress (Rouwette et al., 2011) and ACTH was significantly increased 30 min after cage switch (Morimoto et al., 1991). Thus, the present results after 90 min of NES support the hypothesis of a prolonged corticosterone response in aged rats while corticosetrone already reached basal levels in young animals, which, again might be linked to some resistance to negative feedback mechanisms in the aged. Indeed, for example, suppressive effects of dexamethasone injection on plasma corticosterone were lost in aged rats and to some extent in humans of old age (Wang et al., 1997; Mizoguchi et al., 2009). Such a scenario, however, does not explain the lack of NES-induced CRH-expression in the aged. Therefore, additional mechanisms might contribute to the prolonged NES-hyperthermic response in old rats.

Some previous studies suggest that increased body core temperature in response to cage-switch is rather a fever than hyperthermia. As such, a few studies have shown that plasma IL-6 levels do increase by NES (LeMay et al., 1990; Soszynski et al., 1996) and that the response in the brain is PGE2 dependent using COX inhibitors in an open field stress experiment (Singer et al., 1986; Kluger et al., 1987) or cage switch (Morimoto et al., 1991). Recently, acute 30 min restraint stress as well proved to increase plasma IL-6 and brain COX2 (Serrats et al., 2017). In addition, a glucocorticoid antagonist RU38486 enhanced body core temperature in animals exposed to an open field when injected systemically (Morrow et al., 1993) or i.c.v. (McClellan et al., 1994) again suggesting that old rats may be resistant to glucocorticoid action on thermogenesis during NES. Indeed, we have previously shown that circulating IL-6 induces COX2 (Rummel et al., 2006) and its downstream terminal enzyme mPGES (Rummel et al., 2011) in the hypothalamus and fever using systemic IL-6 injection or an IL-6 antiserum during fever induced by local LPS-induced inflammation. Recently, this observation has been confirmed using genetically modified animals demonstrating an IL-6 activated PGE2-dependent fever induction via brain endothelial cells (Eskilsson et al., 2014). However, the pyrogenic activity of IL-6 is rather weak (Harre et al., 2002; Rummel et al., 2006) and needs to reach a relatively high threshold to activate brain cells and induce fever (Rummel et al., 2004). This threshold can be reduced when IL-6 is combined with low doses of other cytokines such as IL-1 (Cartmell et al., 2000). Interestingly, low circulating IL-6 levels that also occur during exercise seem to correlate with COX2 expression in the hypothalamus (Kruger et al., 2016) but are not accompanied by fever.

The social defeat paradigm and other models of stress-induced increase in body core temperature, which are not PGE2-dependant, clearly differ from fever in underlying brain circuits responsible for vasoconstriction and brown adipose tissue thermogenesis (Nakamura, 2015). While fever is induced via PGE2-stimulated activation of EP3 receptors in the median preoptic nucleus (Lazarus et al., 2007), stress-induced increase in body core temperature in social defeat involves a monosynaptic pathway of glutamatergic activation of the rostral medullar raphe sympathetic premotor neurons (Kataoka et al., 2014), which is not altered by PGE2 synthesis inhibitors (Lkhagvasuren et al., 2011). Similarly, another model of combined stress using i.p. injections followed 1 h later by a novel cage switch did also not reveal any effects of the COX2 inhibitor aspirin on stress-induced rises in body temperature (Vinkers et al., 2009). In the present study, neither plasma IL-6 levels nor COX2- and mPGES-mRNA expression were altered by NES or age supporting that the observed stress-response in this setting represents hyperthermia and not a PGE2-dependent fever. Oka et al. (2001) proposed that PGE2-dependence of psychological stress induced increases in body core temperature might be related to the anticipatory nature of the stress stimulus (Oka et al., 2001).

Apparent discrepancies between the studies that show or do not show effects of COX-inhibitors on the hyperthermic/fever-like responses seem to partially pertain to the intensity of the stress model, e.g., combined or repeated stressors as compared to more moderate stressors such as open filed and cage switch alone. For example, indomethacin was injected 5 h prior to the open field stress by Kluger et al. (1987) as opposed to injection of aspirin 1 h before the NES by Vinkers et al. (2009). In addition, the specific COX-inhibitor type and dose, and modest differences in stress-induced glucocorticoid levels between strains of mice (Romeo et al., 2013) as well as variance in responsiveness to stress between strains of rats might play a role (Porterfield et al., 2011). For instance, it has been shown that Fisher rats are more sensitive than Sprague-Dawley rats to restraint stress-induced IL-1 in the brain (Porterfield et al., 2011) or chronic mild stress-induced plasma glucocorticoid levels (Wu and Wang, 2010) and show higher HPA-axis activity compared to Lewis rat strains (Sternberg et al., 1992; Moncek et al., 2001). Another study demonstrated that some mouse strains like BALB/C mice are more sensitive to CRH antagonist treatment than others (Conti et al., 1994).

As readouts of brain cell activation and inflammation in the brain we used NF-IL6 immunohistochemistry and revealed differences in NF-IL6 activation patterns between young and aged rats. NF-IL6 can be activated by inflammatory (Ejarque-Ortiz et al., 2007; Damm et al., 2011) or psychological stimuli (Fuchs et al., 2013) and regulates the expression of several target genes including IL-6 (Akira et al., 1990). Moreover, NF-IL6-expression and protein levels are increased in the aged brain (Sandhir and Berman, 2010; Koenig et al., 2014). Our present results of increased hypothalamic IL-6-mRNA expression in the aged rats are in line with our earlier results (Rummel et al., 2004) and the inflammatory status, which develops in the brain during aging (Cartmell et al., 2000). Previously, we detected NF-IL6-activation in brain structures of young rats involved in the HPA-axis, namely, the pituitary or the PVN and the pituitary after LPS or cage switch, respectively (Damm et al., 2011; Fuchs et al., 2013) and revealed that NF-IL6 is involved in regulation of basal HPA-axis activity using NF-IL6 deficient mice (Papadimitriou and Priftis, 2009). Now, for the first time, we also found NES-induced activation of the pituitary but not of the PVN in aged rats. Interestingly, the absence of NES-induced NF-IL6 activation in the PVN, which we previously observed in young rats (Fuchs et al., 2013), is in line with the observed lack of increased CRH mRNA expressing after NES in the old rats as this transcription factor can trigger CRH promotor activity (Stephanou et al., 1997).

When using the inflammatory stimulus LPS, we discovered a moderate NF-IL6-activation in the ME, which was significantly stronger in the aged rats 24 h after stimulation. We recently hypothesized a role for ME NF-IL6 activity in potentially gating HPA-axis activity as CRH is expressed in the PVN and released into the ME to trigger ACTH-production and its release from the pituitary (Papadimitriou and Priftis, 2009; Damm et al., 2011). Thus, enhanced LPS-stimulated NF-IL6-activation in the ME of aged rats most likely contributes to dysregulation of the stress-axis during aging. In addition, LPS-induced pituitary activation was more pronounced in the posterior pituitary lobe of aged compared to young rats. Importantly, some of these NF-IL6-positive cells were CD68-positive immune cells but not pituicytes. Moreover, we observed higher numbers of these immune cells in the posterior lobe of the aged rat pituitary potentially signifying some recruitment of activated immune cells. In the brain, CD68 stains activated microglia or perivascular cells (Graeber et al., 1989; Damm et al., 2011). An increase in phagocytosis activity has previously been proposed after LPS-stimulation for the posterior lobe of the pituitary and was also detected by higher CD68 immunoreactivity (Watt and Paden, 2001). However, the enhanced staining of CD68-positive cells in old compared to young rats, which were as well partially NF-IL6-activated, has not been reported before and might represent another mechanism how the brain is targeted by a pro-inflammatory status during aging. Potential immune-cell recruitment is in line with the fact that the posterior pituitary lobe belongs to the circumventricular organs, brain structures with a leaky blood-brain barrier/interface, prone to interact with circulating immune cells and mediators (Roth et al., 2004). As such, perivascular cells contribute to HPA-axis activation during restraint stress as recently revealed by Serrats et al. (2017) using clodronate liposomes to deplete this cell population (Serrats et al., 2017). Thus, these cell types might as well modulate the function of the posterior pituitary lobe but the exact physiological significance remains to be further investigated. So far, some influence of vasopressin together with CRH for the stimulation of ACTH-release in the anterior pituitary lobe has been suggested and might play some role (Papadimitriou and Priftis, 2009). Otherwise, it is well known that vasopressin regulates blood pressure and water homeostasis in the kidney potentially linked to deficits in water intake and water homeostasis such as high levels of vasopressin and lack of thirst that occur during aging in humans (Begg, 2017). Indeed, water intake is reduced in aged rats using the same dose of LPS (Koenig et al., 2014) and most likely accompanied by high vasopressin levels.

In addition, local cytokine action within the pituitary is also known to contribute to HPA-axis activity regulation and NF-IL6 seems to partially contribute to the regulation of their expression (Fuchs et al., 2013). For example, IL-6 is involved in LPS-induced ACTH secretion via paracrine signaling in the pituitary (Gloddek et al., 2001) while TNFα might act stimulatory (Milenkovic et al., 1989) or inhibitory (Gaillard et al., 1990) on the HPA-axis (Fuchs et al., 2013). Within the so-called cytokine cascade, TNFα can induce IL-6 expression (Van Damme et al., 1987; Zhang et al., 1990). Conversely, IL-6 is known to exert a negatively feedback effect on TNFα production (Schindler et al., 1990) and the anti-inflammatory cytokine IL-10 inhibits expression of both cytokines (Strle et al., 2001). Here, we aimed to investigate potential differences in the paracrine pituitary cytokine network between young and old rats. For this purpose we applied purified antisera against the rat IL-6 and IL-10 on primary LPS-stimulated cell cultures of the anterior pituitary lobe and found for young rats that both cytokine inhibition strategies significantly enhanced LPS-induced TNFα secretion from the anterior pituitary. These results can be explained by afore mentioned inhibitory effects of both cytokines on TNFα production (Schindler et al., 1990; Strle et al., 2001). As expected, IL-6 antiserum significantly reduced IL-6 bioactivity. Interestingly, IL-10 antiserum also reduced IL-6 bioactivity potentially suggesting some overlap of the antiserum on IL-6 in addition to binding to IL-10. Reduced IL-6 bioactivity enhanced LPS-induced TNFα levels with the disappearance of its negative feedback on TNFα. In aged animals, LPS also increased IL-6 and TNF bioactivity in the supernatants but neither IL-6 nor IL-10 antiserum significantly altered the cytokine levels. Overall, these results suggest some deficits that seem to evolve in aged anterior pituitary cells for the cytokine network; i.e., IL-6 levels could not significantly be inhibited by the antisera. Subsequently, TNFα levels were not affected. Thus, local paracrine action of cytokines in the aged pituitary might not be sufficient to adjust appropriate function and might contribute to the overall differences of HPA-axis activity between young and old.

### Clinical perspective

Mixed results have been revealed concerning the impact of aging on the HPA-axis activity in humans (Gaffey et al., 2016). Gaffey et al. (2016) overall conclude that the diurnal rhythm is maintained during aging while cortisol output increases with age. Such a scenario is most likely related to a loss or weakening of the negative feedback loop via glucocorticoid receptors in the brain. Thus, acute challenges result in higher plasma cortisol levels in the old age (Otte et al., 2005), similar to what we observed in the present study in rodents to NES-stress. However, in humans, longitudinal studies will be necessary to get more insights into the altered function of HPA-axis in relation to aging, stress/individual health status and risk to develop disease (Gaffey et al., 2016).

Human diversity in stress response and resilience might show similarities to described species and strain dependant differences as discussed in detail above. A recent study by Rimmerman et al. (2017) using fractalkine deficient mice suggests a role for a differential transcriptome due to fractalkine in hippocampal microglia to convey stress resilience (Rimmerman et al., 2017). Future studies will help to reveal if such mechanisms might also be functional and important for stress resilience in humans.

Overall, rodent models are important to reveal insights into species conserved and species specific underlying mechanisms in evolutionary well conserved responses like stress including stress-induced hyperthermia. In addition to previous advances in brain intrinsic neuronal circuits that explain thermogenesis to psychological stress (Nakamura, 2015), we have added to the current understanding on how aging might affect hypothalamic and pituitary activation patterns and cytokine networks. “Psychogenic fever” is known to occur in humans (Nakamura, 2015) and psychological stress seems to contribute to such processes as for example reported in a recent case report (Oka et al., 2013). Further studies will be necessary in humans and animals to gain more insights into underlying mechanisms that contribute to alteration in the HPA-axis and related fields such as loss of thirst and so-called “psychogenic fever” in old age.

## Data availability statement

Datasets are available on request.

## Author contributions

SK, JR, and CR contributed to the conception and design of the study. *In vivo* experiments and sample preparation were conducted by SK, JB, and CR. Cell culture experiments were performed by SK and FF. SK performed statistical analyses. Data analyses and interpretation were done by SK, JB, and CR. SK, JB, AP, JR, and CR contributed to writing the article and to revising the content. All authors proofread the final manuscript.

### Conflict of interest statement

The authors declare that the research was conducted in the absence of any commercial or financial relationships that could be construed as a potential conflict of interest.
