# Supplementary figures and images for: Age Dependent Hypothalamic and Pituitary Responses to Novel Environment Stress or Lipopolysaccharide in Rats
Source: Front Behav Neurosci. 2018 Mar 19;12:55. doi: 10.3389/fnbeh.2018.00055 (PMC5868128; doi:10.3389/fnbeh.2018.00055)

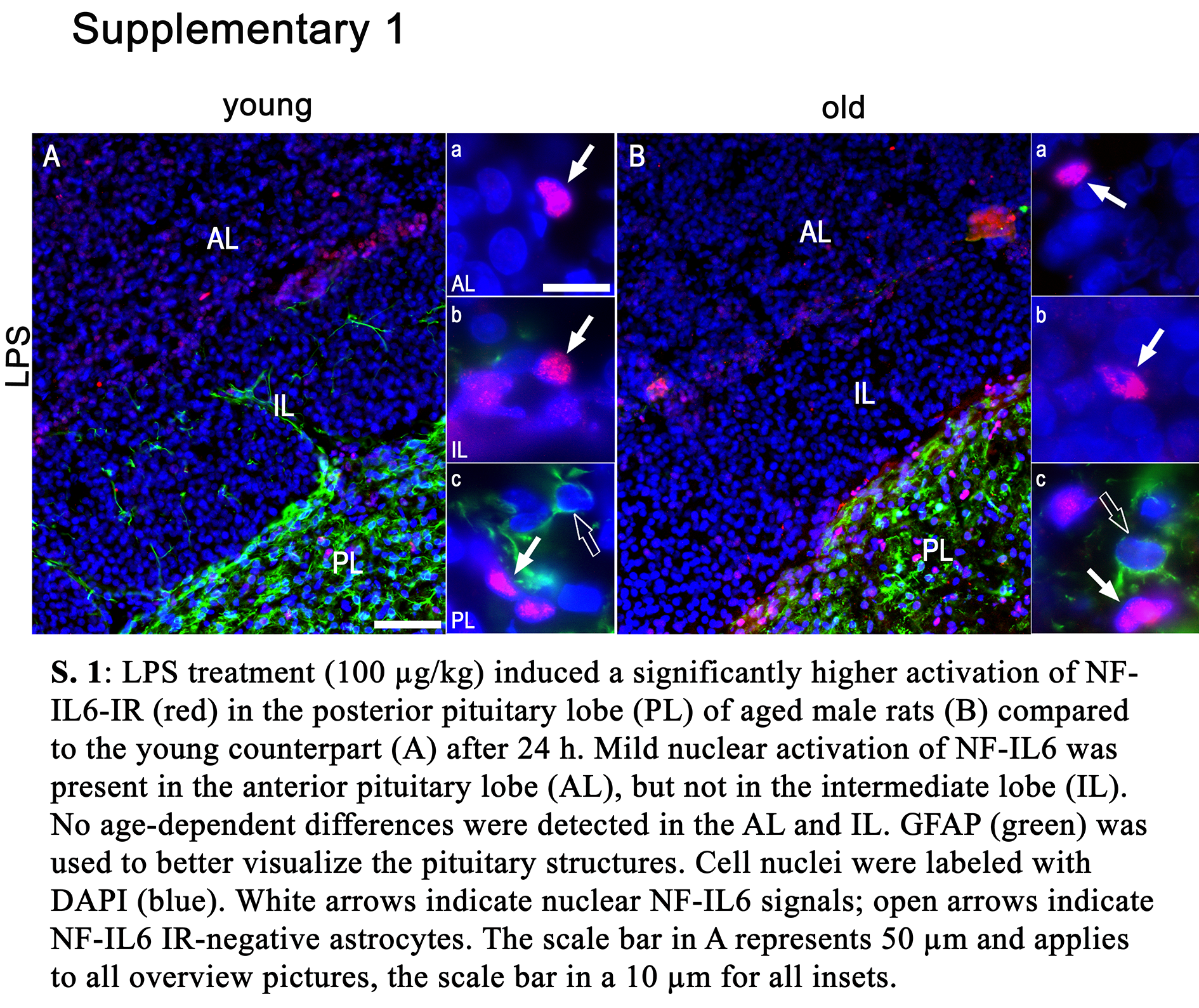

Supplement: Supplementary file 1 [file Image1.TIF]

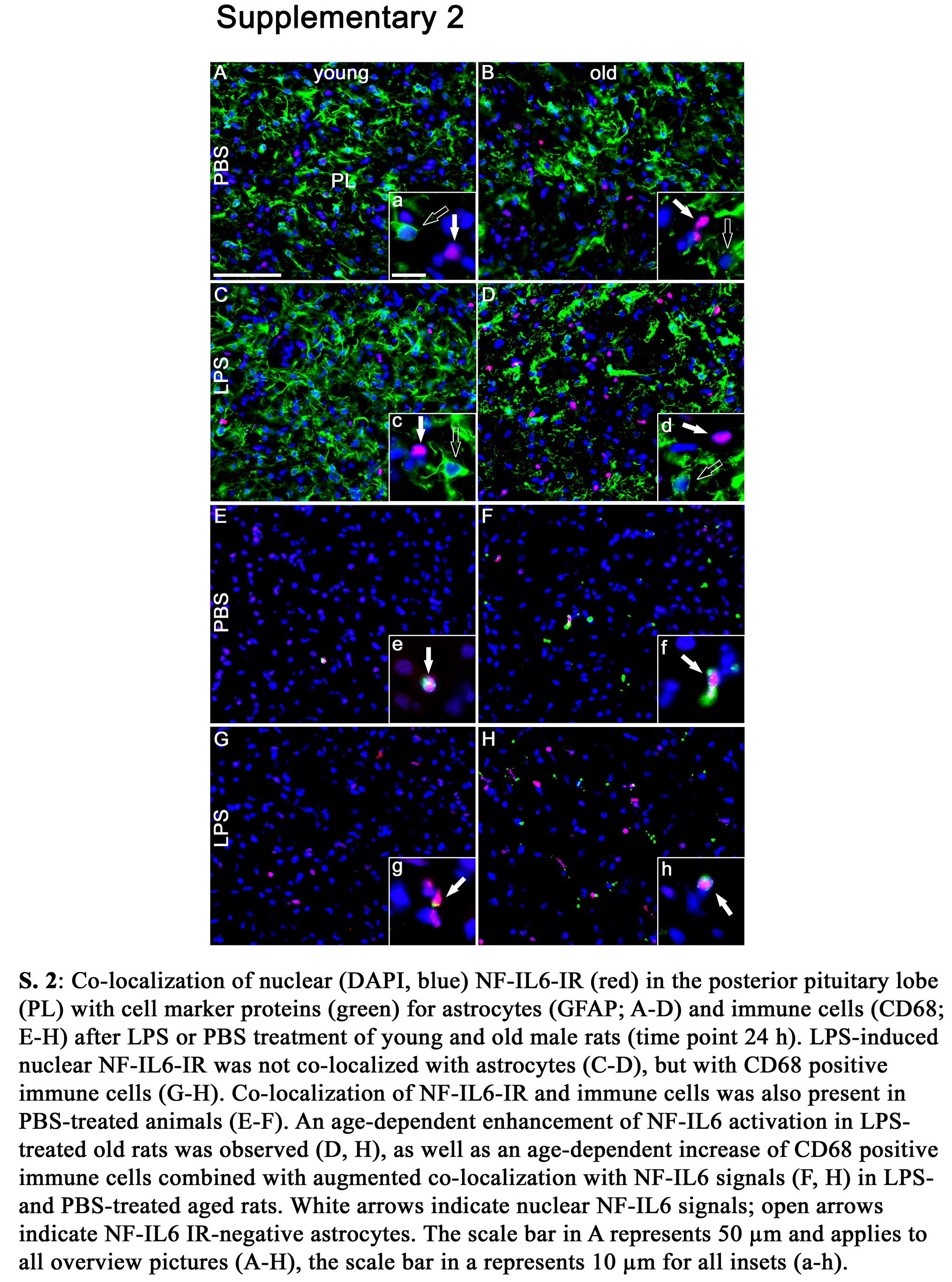

Supplement: Supplementary file 2 [file Image2.TIF]
